# Genomic replacement of native *Cobitis lutheri* with introduced *C. tetralineata* through a hybrid swarm following the artificial connection of river systems

**DOI:** 10.1002/ece3.1027

**Published:** 2014-03-24

**Authors:** Ye-Seul Kwan, Myeong-Hun Ko, Yong-Jin Won

**Affiliations:** 1 Division of EcoScience, Ewha Womans University52, Ewhayeodae-gil, Seodaemun-gu, Seoul, Korea; 2 Department of Life Science, Ewha Womans University52, Ewhayeodae-gil, Seodaemun-gu, Seoul, Korea

**Keywords:** Artificial river connection, *Cobitis lutheri*, *Cobitis tetralineata*, genetic replacement, hybrid swarm, introduced non-native species

## Abstract

River connections via artificial canals will bring about secondary contacts between previously isolated fish species. Here, we present a genetic consequence of such a secondary contact between *Cobitis* fish species, *C. lutheri* in the Dongjin River, and *C. tetralineata* in the Seomjin River in Korea. The construction of water canals about 80 years ago has unidirectionally introduced *C. tetralineata* into the native habitat of *C. lutheri*, and then these species have hybridized in the main stream section of the Dongjin River. According to the divergence population genetic analyses of DNA sequence data, the two species diverged about 3.3 million years ago, which is interestingly coincident with the unprecedented paleoceanographic change that caused isolations of the paleo-river systems in northeast Asia due to sea-level changes around the late Pliocene. Multilocus genotypic data of nine microsatellites and three nuclear loci revealed an extensively admixed structure in the hybrid zone with a high proportion of various post-F1 hybrids. Surprisingly, pure native *C. lutheri* was absent in the hybrid zone in contrast to the 7% of pure *C. tetralineata*. Such a biased proportion must have resulted from the dominant influence of continually introducing *C. tetralineata* on the native *C. lutheri* which has no supply of natives from other tributaries to the hybrid zone due to numerous low-head dams. In addition, mating experiments indicated that there is no discernible reproductive isolation between them. All the results suggest that the gene pool of native *C. lutheri* is being rapidly replaced by that of continually introducing *C. tetralineata* through a hybrid swarm for the last 80 years, which will ultimately lead to the genomic extinction of natives in this hybrid zone.

## Introduction

A number of hybridizations have been reported across a wide taxonomic range of animal species (Vrijenhoek et al. 1989; Schwenk et al. 2008). This is also the case in fish species and several factors such as weak behavioral isolation, and limited available spawning habitats have been attributed to frequent events of hybridization of fish species (Scribner et al. 2001; Gozlan et al. 2010).

According to recent evidences, hybridizations have been recognized as an important factor in the evolution of populations and species, influencing genetic variation, functional novelties, adaptive radiation, and speciation in a number of animal groups, including cichlid fish (Barton 2001; Seehausen 2004; Mallet 2007; Abbott et al. 2013). However, in a conservation context, hybridizations between distinct species are related to negative effects on the fitness of parental species, which can even cause extinction (Rhymer and Simberloff 1996; Allendorf et al. 2001, 2010; Vitule et al. 2009). In particular, interspecific hybridization with introgression between closely related species can create a hybrid swarm in which the unique genetic characteristics of the parental species are entirely admixed (Allendorf et al. 2001; Taylor et al. 2006; Crispo et al. 2011; Stemshorn et al. 2011; Winkler et al. 2011). A hybrid swarm has the potential to decrease taxonomic diversity through genetic extinction, causing the loss of monophyletic taxa (Epifanio and Philipp 2001). For this reason, hybridization and introgression raise concerns about species conservation and diversity, as human-induced introductions and invasions of non-native species have increased (Allendorf et al. 2001; Epifanio and Philipp 2001; Crispo et al. 2011).

**Figure 1:**
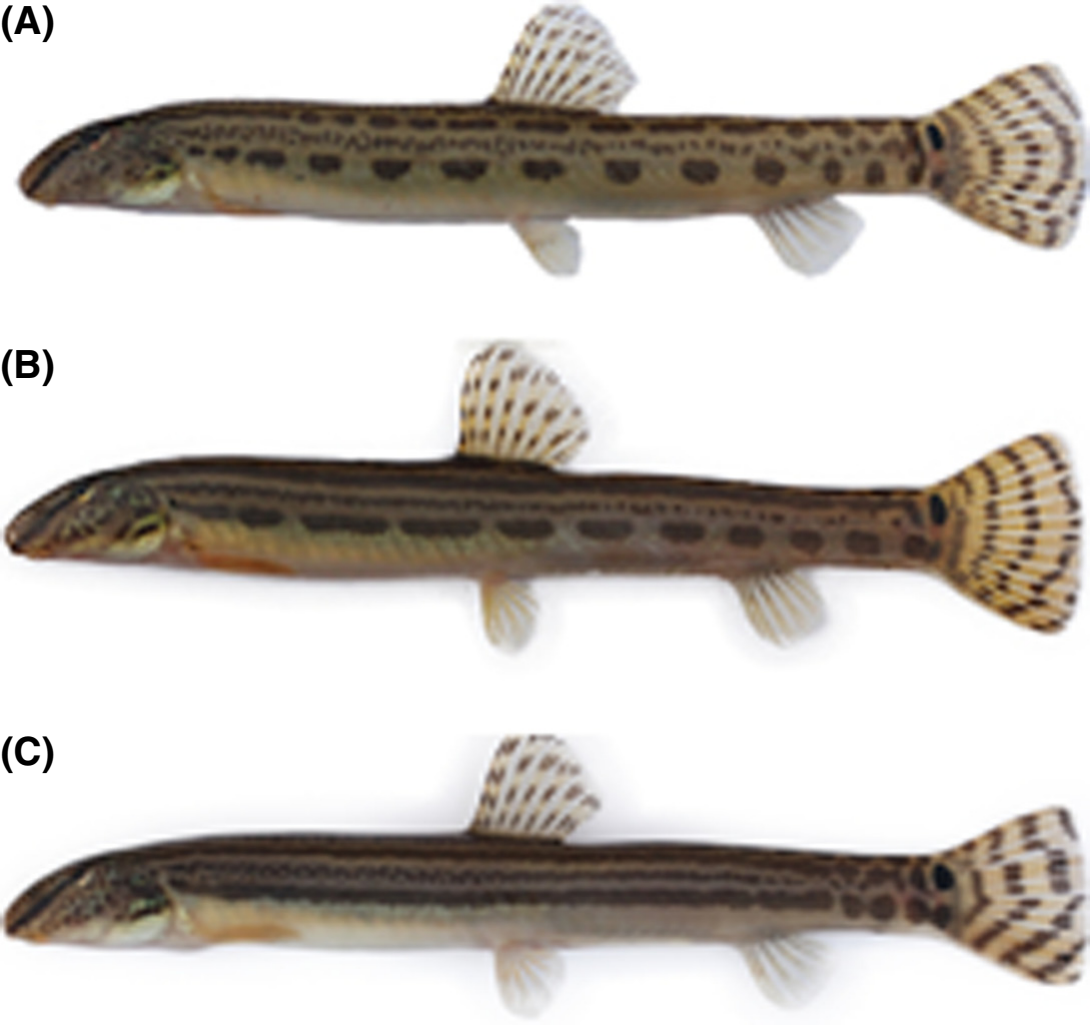
Difference of the color pattern of (A) a pure *Cobitis lutheri*, (B) a hybrid between *C. lutheri* and *C. tetralineata*, and (C) a pure *C. tetralineata*.

The freshwater fish of the genus of *Cobitis* (Cypriniformes, Cobitidae) are well known to undergo natural hybridizations with closely related species (Vasil'ev et al. 1989; Kim and Lee 1990; Kitagawa et al. 2003; Janko et al. 2007). One special case of hybridizations between *Cobitis* species was discovered in the Dongjin River in the southern region of Korea. This belongs to the human-induced hybridization, involving a secondary contact of native *C. lutheri* and introduced non-native *C. tetralineata* from the neighboring Seomjin River following artificial connection of the rivers' tributaries through water canals (Kim and Lee 1984; Kim and Yang 1993). *Cobitis lutheri* Rendahl, 1935, commonly known as the sand spined loach, inhabits the bottoms of rivers and streams across a wide area of northeast Asia, including China, Korea, and Russia (Kim 2009). In Korea, this species inhabits in the rivers flowing westward toward the Yellow Sea between Korea and China. In contrast, the relatively recently described Korean striped spine loach, *C. tetralineata* Kim, Park and Nalbant, 1999, is an endemic species distributed only in the Seomjin River, which flows southward toward the South Sea on the Korean Peninsula (Kim et al. 1999). These two allopatric species are generally distinguished by their body color patterns; *C. lutheri* exhibits rows of quadrate blotches in the second and fourth zones of its body, whereas *C. tetralineata* displays broad stripes from behind the margin of its opercula to the base of its caudal fin (Kim and Lee 1988; Kim 2009) (Fig. 1). However, despite such differences, *C. lutheri* and *C. tetralineata* are considered to be ecologically equivalent species that prefer similar environments, such as the shallow sandy bottoms of river tributaries (Kim and Lee 1988; Kim 2009). In addition, males of the two *Cobitis* species exhibit a similar appearance during their overlapping spawning seasons, from June to July (Kim et al. 2006; Ko and Park 2011).

Intensive hybridization between these two allopatric species began in the Dongjin River following the construction of two canals for hydroelectric power generation and irrigation from Lake Okjeong in the upper part of the Seomjin River to the Dongjin River in 1931 and 1945 (Kim and Lee 1984). Although these two distinct rivers are connected by the canals, the inflow of water occurs only in one direction, from the Seomjin River to the Dongjin River, because of the difference in altitude between the connected points of the rivers. For this reason, *C. tetralineata* has continuously entered the Dongjin River through the canals with the water flow from the Seomjin River for 80 years, without the reverse migration of *C. lutheri* to the Seomjin River. As a result of these continuing introductions, individuals with intermediate morphological characteristics between the native *C. lutheri* and the introduced *C. tetralineata* have been frequently observed, indicating widespread intergradation between them (Kim and Lee 1984). In a previous allozyme study that compared the allelic and morphological patterns of these hybrids, it was suggested that both interbreeding between the pure parental species and backcrossing from the F1 generation have occurred in the Dongjin River hybrid zone (Kim and Yang 1993). In this hybrid zone, the problem of the taxonomic classification of the two species was a central issue attracting taxonomists' interests (Kim and Lee 1984, 1988; Kim and Yang 1993). However, the consequences of the human-induced hybridization for 80 years have rarely been addressed with regard to the potential fates of the hybrid populations, including the formation of new lineages and the complete loss of native species.

In the present study, we aim to characterize the hybridizing populations in the Dongjin River with molecular population genetic tools and to anticipate its fate particularly in relation to the currently continuing introduction of the non-native *C. tetralineata* through two canals from the Seomjin River. For this purpose, we genetically examined both the nonhybridized populations of two *Cobitis* species from allopatric localities and the hybrid populations. In parallel, artificial mating experiments were performed to verify the reproductive capacity of hybrids with various genetic backgrounds and thus the future consequences of the ongoing hybridization on the native *Cobitis* fish in the Dongjin River.

## Materials and Methods

### Sampling and DNA extraction

**Figure 2:**
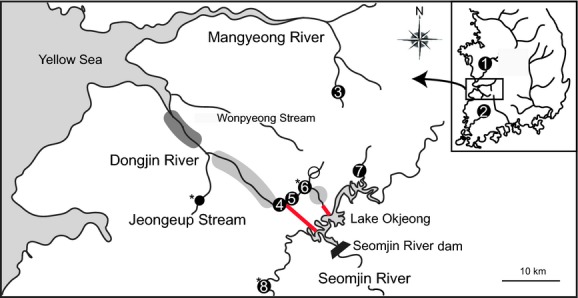
Map of sampling localities. Circles with numbers indicate sampling localities and sampling information of each locality was presented in Table 1. The red lines represent two canals connecting the waters of the Dongjin River and Lake Okjeong. Empty circle indicates localities where no *Cobitis* individuals were observed. Dark and light gray shadings indicate areas which were not included in the present study due to brackish water and high water level with strong current, respectively. Asterisks mark the sampling sites where mature fish individuals were collected for mating experiments.

A total of 171 *Cobitis* individuals were sampled from eight localities in the autumn of 2007 and the summer of 2010. Each sampling site and the number of samples collected are shown in Fig. 2 and Table 1. To obtain comparative genetic information on the nonhybridized species from adjacent rivers where only single species distributes in allopatry, *C. lutheri* samples were collected from three river systems (locality 1, 2, and 3 in Fig. 2). Sample collection was also attempted in a tributary of the Dongjin River, the Jeongeup Stream, where morphologically pure *C. lutheri* was reported (Kim and Lee 1984). However, this population was excluded from the present genetic analyses, because very few *Cobitis* individuals were obtained from the tributary during the current study period due to recent habitat alterations by dredging construction. *C. tetralineata* was sampled in two different tributaries of the Seomjin River, the Oknyeodong Stream and the Churyeong Stream (locality 7 and 8). In the Dongjin River, possible habitats were investigated along the mainstream of the river, where the hybrid individuals were previously observed (Kim and Lee 1984; Kim and Yang 1993). The area of dark shading in Fig. 2 was excluded in our investigation because this area is brackish not suitable for *Cobitis* fish. During the study period, sample collection was impossible in the area of light shading (Fig. 2) because of strong water flows and high water level under the influence of discharged water from the canals. Thus, final sample collection for the population genetic analyses was limited to the very short section of the main stream at three sites (locality 4, 5, and 6 in Fig. 2). Genomic DNA was extracted from the muscle tissue of each specimen using the Qiagen Tissue and Blood kit (Qiagen, Hilden, Germany) following the manufacturer's protocols.

**Table 1:** List of specimens, locality information, and the number of specimens (*N*) examined for genetic analyses.

Taxon	Locality (county, province), locality number	GPS	*N*
*Cobitis lutheri*	Geum River (Gongju, Chungcheongnamdo), 1	36°27′N, 127°04′E	8
*C. lutheri*	Youngsan River (Damyang, Jeollanamdo), 2	35°17′N, 127°02′E	7
*C. lutheri*	Mangyeong River (Jeonju, Jeollabukdo), 3	35°47′N, 127°11′E	30
*C. lutheri*	Jeongeup Stream (Jeongeup, Jeollabukdo)^2^	35°34′N, 126°49′E	–
*C. lutheri* × *C. tetralineata*^1^	Dongjin River (Chilbo, Jeollabukdo), 4	35°36′N, 126°59′E	13
*C. lutheri* × *C. tetralineata*^1^	Dongjin River (Chilbo, Jeollabukdo), 5	35°36′N, 127°00′E	59
*C. lutheri* × *C. tetralineata*^1^	Dongjin River (Sanoe, Jeollabukdo), 6	35°37′N, 127°02′E	11
*C. tetralineata*	Oknyeodong Stream (Imsil, Jeollabukdo), 7	35°40′N, 127°08′E	14
*C. tetralineata*	Churyeong Stream (Bokeung, Jeollabukdo), 8	35°26′N, 126°56′E	29
*Misgurnus anguillicaudatus*	Ungcheon Stream (Ungcheon, Chungcheongnamdo)	36°13′N, 126°36′E	2
*M. mizolepis*	Seomjin River (Unbong, Jeollabukdo)	35°26′N, 126°56′E	2
*C. choii*	Kim et al. (2013)	–	1
*Kichulchoia brevifasciata*	Kim et al. (2013)	–	1
*K. multifasciata*	Kim et al. (2013)	–	1
*Koreocobitis rotundicaudata*	Kim et al. (2013)	–	1
*Iksookimia hugowolfeldi*	Kim et al. (2013)	–	1
*I. longicorpa*	Kim et al. (2013)	–	1
*I. pacifica*	Kim et al. (2013)	–	1
*I. koreensis*	Kim et al. (2013)	–	1

1 Hybrid individuals suspected.

2 Sampling sites only for use in mating tests but not for genetics.

### PCR and analysis of DNA sequence data

In the *Cobitis* samples, sequences were obtained from one mitochondrial DNA locus (Cyt *b*, Cytochrome *b* gene) and three nuclear DNA loci (ENC1, similar to ectodermal-neural cortex 1; SH3PX3, similar to SH3 and PX domain-containing three genes; and Ptr, hypothetical protein LOC564097) (Li et al. 2007). PCR amplification of Cyt *b* gene was conducted using the primers, GluDG.L (Palumbi 1996) and H16460 (Perdices and Doadrio 2001). The protocols applied for PCR followed those of Perdices and Doadrio (2001). The sequences of three nuclear DNA were amplified using nested primers, as described in Li et al. (2007), in a 25 *μ*L reaction volume (2 *μ*L of 10X PCR buffer, 1 *μ*L of 2.5 mM dNTPs, 1 *μ*L of 1X bovine serum albumin, 1 *μ*L each of 10 *μ*M primer solutions, 0.12 *μ*L (1 unit) of *Taq* DNA polymerase, 1 *μ*L of genomic DNA, and 12.88 *μ*L of distilled water). Each amplification reaction was performed under the following conditions: initial denaturation at 94°C for 2 min, followed by denaturation at 94°C for 40 sec, annealing at different temperatures depending on the marker (ENCl, 53°C for the first PCR/62°C for the second PCR; Ptr, 55°C/62°C; SH3PX3, 55°C/62°C) for 40 sec, and elongation at 72°C for 1 min, which was repeated for 35 cycles in the first PCR and 30 cycles in the second PCR, with a final elongation step at 72°C for 5 min. All PCR products were sequenced using an ABI 3730xl automatic sequencer (Applied Biosystems, Foster City, CA). To solve the phase problem of heterozygotic nucleotide sites of diploid DNA sequences, DNA sequences were processed with PHASE program (Stephens et al. 2001; Stephens and Donnelly 2003), and all sequences were submitted to GenBank (Cyt *b*, KF661552–KF661720; ENC1, KF702400–KF702745; Ptr, KF702746–KF703091; SH3PX3, KF703092–KF703437).

Parameters related to the population genetic divergence of these sister *Cobitis* species were estimated on the basis of four independent genetic loci and their molecular substitution rates that was calibrated according to a recently discovered fossil from east Asia. The population genetic divergence model IMa2 (Hey 2010) was employed to estimate six population demographic parameters: the effective population sizes, population migration rates per generation (2*Nm*), and splitting time between *C. lutheri* in the Mangyeong River (locality 3) and *C. tetralineata* in the Churyeong Stream (locality 8). To certify whether our data satisfies the assumptions of this IMa2 model, recombination tests of the three nuclear genes were conducted using SITES (Hey and Wakeley 1997). When evidence of recombination was detected in a certain locus, a single largest block of DNA data of the locus without recombinants was finally used for IMa2 analyses (Won and Hey 2005). Additionally, neutrality of each locus was tested using Arlequin win3.5 (Excoffier et al. 2005). Using the obtained maximum prior values for each parameter, the program was independently run five times, with a discarded burn-in of 10,000 steps and a chain of 500 million steps under the infinite site model for three nuclear loci and HKY model for one mitochondrial locus. A heating scheme was employed with 40 chains under a geometric increment model (term 1: 0.975, term 2: 0.750). The generation time of *Cobitis* species was assumed to be 3 years (Ko and Park 2011). Additionally, the likelihood ratio tests of nested demographic models were conducted to statistically compare alternative migration models with the full model based on the estimated genealogies from the MCMC simulation as illustrated in IMa2 manual. To convert the mutation-scaled parameters in the IMa2 analysis into physical years and numbers of individuals, the substitution rates of three markers (Cyt *b*, ENC1, and SH3PX3) were calculated with BEAST (Drummond and Rambaut 2007) using sequences from twelve species of Cobitidae, including *C. lutheri* and *C. tetralineata*. Among the sequences of the three markers, those of eight species (*C. choii*,*Kichulchoia brevifasciata*,*K. multifasciata*,*Koreocobitis rotundicaudata*,*Iksookimia hugowolfeldi*,*I. longicorpa*,*I. pacifica*, and *I. koreensis*) were obtained from Kim et al. (2013). To include complete Cobitidae genera in this analysis, the sequences of two species belonging to the genus *Misgurnus* (*M. anguillicaudatus* and *M. mizolepis*) were also acquired in the present study through PCR amplification following the methods described previously (Kim et al. 2013). Unfortunately, the Ptr gene was excluded in this analysis because it was difficult to obtain clear sequences in some species because of a number of heterozygotic sites. This analysis was conducted separately for each marker with 200 million generations of chains under a model of an uncorrelated lognormal distribution rate as this clock model can provide more accurate clock estimates and possibly true trees (Drummond et al. 2006). A birth–death model was used for a prior tree. The calibration time was given at the ancestral node of the genera *Cobitis*,*Iksookimia*, and *Kichulchoia* as 18 million years ago (MYA), according to the oldest fossil record of *C. longipectoralis*, using a lognormal distribution with a mean of 1.5, a standard deviation of 1.3, and an offset of 17.5 (55.53–18.03 MYA, 95% highest posterior density (HPD))(Chen et al. 2010; Kim et al. 2013).

The whole sequences from the Dongjin River samples were grouped into T-type (originated from *C. tetralineata*) or L-type (from *C. lutheri*). For this grouping, unrooted neighbor-joining trees of haplotypes from the Dongjin River were built by Geneious tree builder (Geneious Pro 6.1.4, Biomatters Ltd.), including haplotypes of the populations of *C. lutheri* and *C. tetralineata* from nonhybridized allopatric localities. Minimum spanning trees of the haplotypes from the Dongjin River were constructed for each DNA marker using Arlequin and HapStar (Teacher and Griffiths 2011). Also, the haplotype frequencies, haplotype diversities (*H*_d_), Tajima's *D* (Tajima 1989a,b1989b), and Fu's *Fs* (Fu 1997) of each marker were calculated using the Arlequin program.

### Genotyping and analysis of microsatellite marker data

To examine the population structure of the hybrids found in the Dongjin River, nine microsatellite markers (*CL*001, *CL*002, *CL*004, *CL*005, *CL*006, *CL*007, *CL*008, *CL*009, and *CL*011) that we developed and characterized in a previous study on *C. lutheri* were used for their high variability (Agata et al. 2011). For the genotyping experiment, PCR amplifications were conducted under the following conditions: 94°C for 5 min; followed by 30 cycles at 94°C for 30 sec, 50°C for 1 min, and 72°C for 1 min; with a final extension at 72°C for 7 min. PCR was performed in a volume of 10 *μ*L containing 1X PCR buffer, 0.2 nmol dNTPs, 1X bovine serum albumin, 5 pmol of the designed primers, 2 pmol of IRD-700 labeled primers (LI-COR, Lincoln, NE), 0.05 U of *Taq* DNA polymerase, and 10–50 ng of template. The samples were genotyped using a model 4300 automatic sequencer (LI-COR). To improve the resolution of genotypic analysis, the haplotype data of the three nuclear genes (ENC1, Ptr, SH3PX3) were included after converting them into multilocus genotype data using PGDSpider (Lischer and Excoffier 2012) under the confirmed neutrality of each locus (Table S1).

Based on the *Cobitis* genotyping data, the population genetic structure was assessed and the proportions of admixture (*q*-values) were calculated using STRUCTURE (Hubisz et al. 2009). A series of priori assumptions was tested regarding the number of populations (*K*) with the scale of the species (*K *=* *2) and with the scale of the populations (*K *=* *3–6). Additionally, pairwise *F*_ST_ was calculated among the nonhybridized allopatric populations (locality 1, 2, and 3 for *C. lutheri*; locality 7 and 8 for *C. tetralineata*) based on the multilocus genotypic data using Arlequin program. To identify various classes of hybrids (F1, F2, and backcrosses) in the Dongjin River, the probability of each distinct hybrid class for each individual was calculated through one million sweeps using NewHybrids, under the framework of Bayesian model-based clustering via Markov chain Monte Carlo simulation (Anderson and Thompson 2002). After classification, the relationship between each hybrid classes and the type of Cyt *b* gene was statistically evaluated by the chi-square test using SPSS version 21.0 (SPSS Inc., Chicago, IL).

### Mating tests

Sexually mature *Cobitis* individuals were obtained in their mating season to investigate the reproductive abilities of hybrid individuals. Pure *C. lutheri*, pure *C. tetralineata*, and *C. lutheri–C. tetralineata* hybrids were, respectively, collected from the Jeongeup Stream of the Dongjin River, the Churyeong Stream of the Seomjin River, and the main Dongjin River in the locality of Sanoe-myeon, based on their morphological characteristics (Figs. 1, 2). Four experimental groups were set up involving the following mating pairs: (1) female *C. tetralineata* and male *C. lutheri* (TT ♀× LL ♂), (2) female *C. lutheri* and male *C. tetralineata* (LL ♀× TT ♂), (3) female hybrids and male *C. tetralineata* (TL ♀× TT ♂), and (4) female hybrids and male *C. lutheri* (TL ♀× LL ♂). Two additional mating pair groups were tested as controls: (1) female and male *C. tetralineata* (TT ♀× TT ♂), and (2) female and male *C. lutheri* (LL ♀× LL ♂). To obtain completely mature eggs and sperms for artificial fertilization, Ovarprim (Syndel, Qualicum Beach, BC, Canada) was injected into both male and female adult fish. Twelve hours after the injections, mature eggs and sperms were obtained from females and males, respectively, by pressing their abdomens. The eggs were artificially fertilized and bred by spreading sperms in Petri dishes of 15-cm diameter incubated at 25°C. Subsequently, the development of the embryos was observed and photographs of the developmental process were acquired using a dissecting microscope (LEICA S8APO, Wetzlar, Germany) with a digital camera (LEICA DFC290). In addition, six indices of early life history characteristics were measured for the six mating pairs: mature eggs size, swelling eggs size, hatching larvae size, hatching rates, abnormality rates, and early survival rates. The rates of abnormality and early survival were determined 5 days after the hatching of the larvae. The values of each index were statistically compared among the mating groups by analysis of variance (ANOVA) and Tukey's HSD tests using SPSS version 21.0 (SPSS Inc.).

## Results

### Genetic divergence parameters for *C. lutheri* and *C. tetralineata*

**Figure 3:**
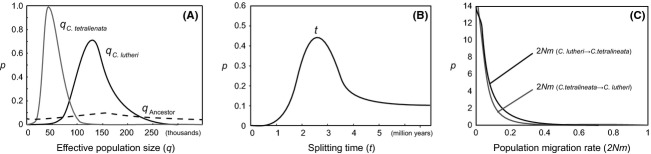
Marginal posterior probability, *P*, of (A) effective population sizes (*q*), (B) the splitting time (*t*) in years between *C. lutheri* and *C. tetralineata*, and (C) population migration rates (2*Nm*) estimated by IMa2.

To infer the divergence process of the allopatric *C. lutheri* and *C. tetralineata*, we estimated population demographic parameters for the Mangyeong and Seomjin River populations (Fig. 3). We used 278 bp of ENC1, 554 bp of Ptr, and 240 bp of SH3PX3 for this analysis after excluding recombinant blocks of DNA segments. The substitution rates (substitutions/site/million years) at each locus calculated using BEAST (Table 2) were employed as mutation rates for the IMa2 analysis. The IMa2 analysis resulted in an estimate of the effective population size of *C. lutheri* in the Mangyeong River (mean of 140,000 with 95% confidence interval (CI) of 82,000–218,000) that was almost three times higher than that of *C. tetralineata* (mean of 42,000 with 95% CI of 20,000–76,000). The splitting time between *C. lutheri* and *C. tetralineata* was estimated to be approximately 3.3 MYA (95% CI of 0.9–6.6). The population migration rates per generation (2*Nm*) between *C. lutheri* and *C. tetralineata* were very close to zero (from *C. lutheri* to *C. tetralineata*, mean of 0.056 with 95% CI of 0.001–0.202; from *C. tetralineata* to *C. lutheri,* mean of 0.053 with 95% CI of 0.002–0.167). The full model of five demographic parameters (*θ*_1_ *θ*_2_ *θ*_A_ *m*_1_ *m*_2_) was not rejected by the three nested models (*θ*_1_ *θ*_2_ *θ*_A_ *m*_1_ = 0 *m*_2_; *θ*_1_ *θ*_2_ *θ*_A_ *m*_1_ *m*_2_ = 0; and *θ*_1_ *θ*_2_ *θ*_A_ *m*_1_ = *m*_2_). All the *P-*values were near one for each alternative nested model.

**Table 2:** Haplotype information for four genes in the hybrid *Cobitis* populations of the Dongjin River.

Locus	Sequence length (bp)	Mutation rates^1^ (95% CI)	Group	Proportion of haplotypes (%)	No. of haplotypes	*H*_d_
Cyt *b* gene	1116	0.0105 (0.0021–0.1295)	T-type	60.49	5	0.5400
			L-type	39.51	10	0.7298
ENC1	788	0.0011 (0.0001–0.0292)	T-type	61.63	7	0.7563
			L-type	38.37	10	0.8196
Ptr	668	–	T-type	65.88	6	0.1541
			L-type	34.12	3	0.4459
SH3PX3	611	0.0016 (0.0001–0.0236)	T-type	53.09	17	0.8525
			L-type	46.91	18	0.8495

CI, Confidence Interval; *H*_d_, haplotype diversity.

1 In units of mutations/site/million years.

### Intermixed haplotype pattern in the hybrid zone

**Figure 4:**
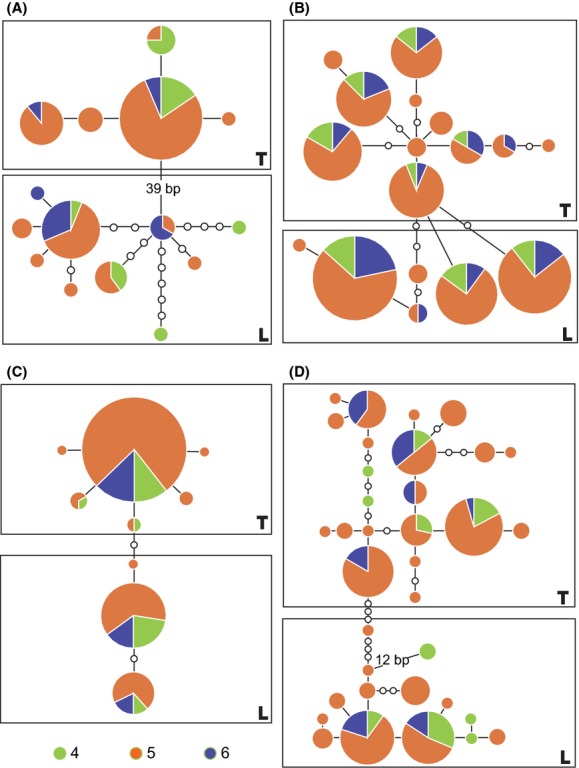
Haplotype parsimony networks for four genes: (A) Cytochrome *b*, (B) ENC1, (C) Ptr, and (D) SH3PX3. The pie charts represent distinctive haplotypes and their frequencies at each sampling site in the Dongjin River. The size of the circles is proportional to the frequency of each haplotype. Empty circles represent hypothetical haplotypes that were not observed in our samples. The rectangular boxes delimit the haplotypes into two groups: the T-type group (*Cobitis tetralineata*) and the L-type group (*C. lutheri*), depending on the genetic distance to their pure allopatric species (Fig. S1). 4, locality number 4; 5, locality number 5; 6, locality number 6 in Table 1.

The genetic pattern of hybrids between *C. lutheri* and *C. tetralineata* in the Dongjin River (locality 4, 5, and 6) was initially investigated with the haplotypes of one mitochondrial and three nuclear genes. The haplotypes of the populations were clearly divided into two groups (L-type and T-type), which represented two genetic lineages corresponding to the pure allopatric species, *C. lutheri* and *C. tetralineata,* respectively (Fig. 4). When we partitioned the haplotypes into the two groups, the haplotype diversity (*H*_d_) of the T-type was relatively lower than that of L-type except for the SH3PX3 gene, although the proportion of T-type was higher than that of L-type at all loci (Table 2). Both Tajima's *D* and Fu's *Fs* values were not significant to reject the assumption of neutral evolution at all the loci of the two allopatric species (Table S1). This neutrality also holds for the introduced T-type group within the hybrid zone except for Ptr gene.

### Admixed population genetic structure and pattern of hybrid classes

**Figure 5:**
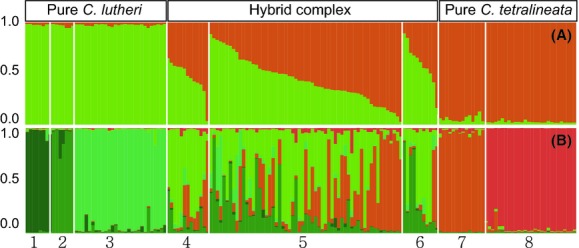
STRUCTURE results for all the sampled individuals of *Cobitis lutheri* and *C. tetralineata* and their hybrids given (A) *K *=* *2 and (B) *K *=* *6 clusters. Numbers under the histogram are locality numbers shown in Table 1.

Analyses of the population genetic structure based on multilocus genotypic data were performed for all the sampled individuals from the hybrid zone as well as the other allopatric localities of *C. lutheri* and *C. tetralineata* from the closest neighboring rivers of the Dongjin River. When we assumed *K* to be equal to two (*K *=* *2) from the perspective of interspecific hybridization, the individuals of the hybrid zone (locality 4, 5 and 6) clearly exhibited variously admixed ancestries between the two species, *C. lutheri* and *C. tetralineata* (Fig. 5A). Each population of the three sites showed a similar gradient pattern of admixture between the two species. The gradient pattern demonstrated an absence of species boundaries but an excessive intergradation between the two parental *Cobitis* species in the hybrid populations.

The structure analyses showed the highest posterior probability under the six-population model (*K *=* *6) among the hypothetical number of populations (*K *=* *2–6), which corresponds to the number of sampled localities representing the investigated rivers or streams (*n *=* *6). This model suggested that the nonhybridized allopatric populations of each species were genetically subdivided into several groups (Fig. 5B). In particular, the two populations from the Churyeong and Oknyeodong Streams representing allopatric *C. tetralineata* of the Seomjin River showed a clear genetic difference, despite the fact that both localities belong to the same river. We confirmed this genetic distinctiveness through significant pairwise *F*_ST_ values among them (Table S2). In the hybrid zone of the Dongjin River, three genetic groups were found to be admixed with each other. One of these groups was identical to the genetic group of *C. lutheri* from locality 2, and another was consistent with the genetic group of *C. tetralineata* from locality 7. The last group was found only in the populations from the Dongjin River.

**Figure 6:**
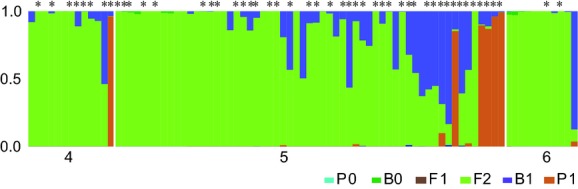
Bar plot of the posterior probabilities of hybrid classes for the individuals from the hybrid zone in the Dongjin River computed by NewHybrids. Numbers under the histogram are locality numbers shown in Table 1. P0, pure *Cobitis lutheri*; B0, backcrosses with pure *C. lutheri*; F1, F1 hybrid; F2, F2 hybrid; B1, backcrosses with pure *C. tetralineata*; P1, pure *C. tetralineata*. Asterisks on the bar plot indicate the mitochondrial Cyt *b* haplotype of *C. tetralineata*. P0, B0, and F1 individuals are absent in the hybrid zone.

After confirming the intensive interspecific hybridization, we characterized the individuals of the Dongjin River by fitting them into the proposed six classes of hybrid after two generations of hybridization using NewHybrids. To minimize misidentification, we used a reliable threshold posterior probability of 0.50 for NewHybrids (Vähä and Primmer 2006; Winkler et al. 2011). The NewHybrids analysis showed that most of individuals from the hybrid zone could be classified as follows: 6 pure *C. tetralineata* (P1 in Fig. 6), 9 backcrossed individuals with *C. tetralineata* (B1) and 68 F2 descendants (F2), with high posterior probabilities. No individuals were identified as being pure *C. lutheri* (P0), backcrossed with *C. lutheri* (B0) or F1 hybrids (F1). The individuals categorized into P1 or B1 were highly corresponded with Cyt *b* gene of *C. tetralineata*, while F2 hybrids were relatively unbiased for the Cyt *b* (chi-square = 8.046, df = 2, *P *=* *0.016, Fig. S2).

### Mating tests

**Figure 7:**
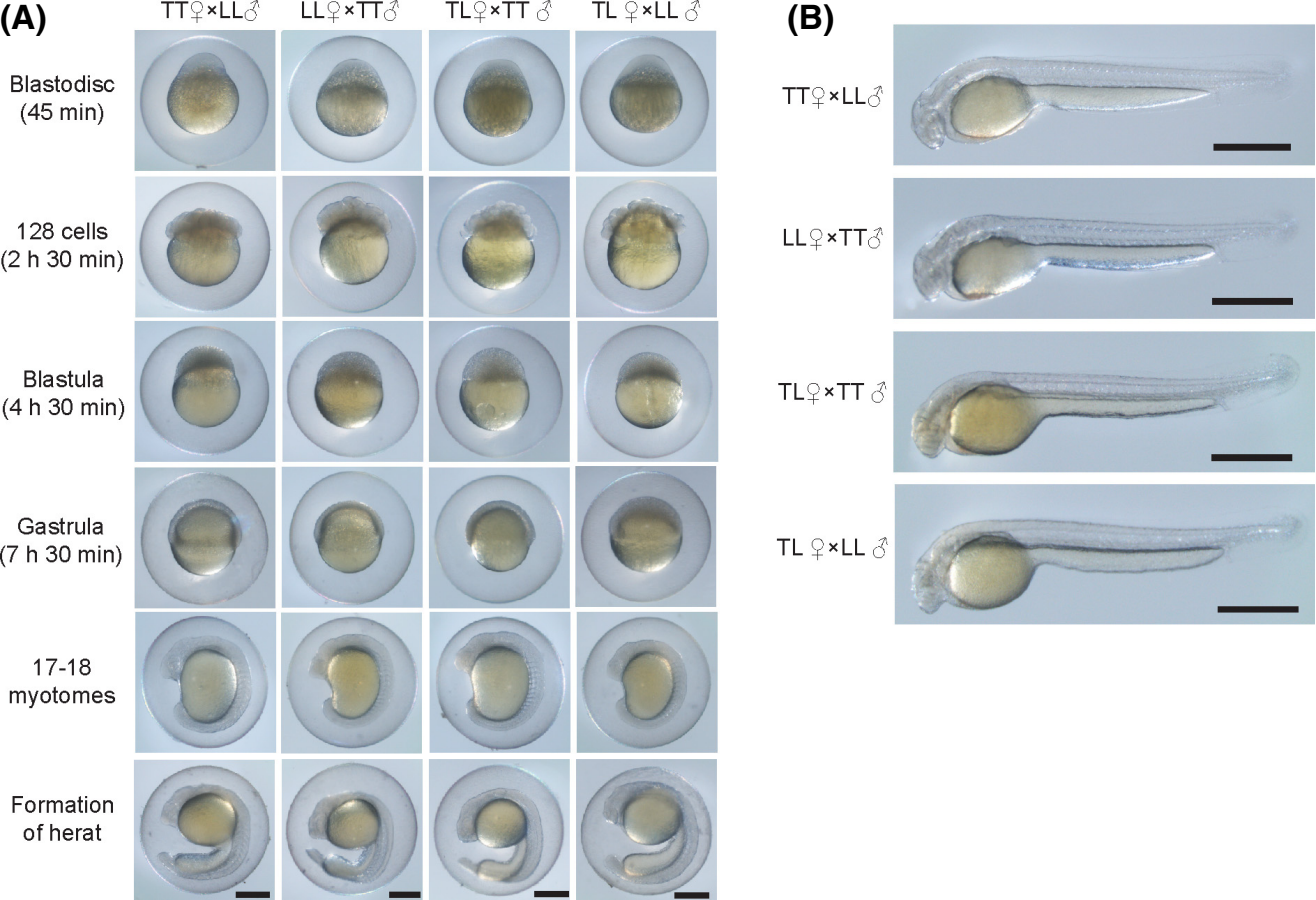
Time-series micrographs of (A) the developmental stages of *Cobitis tetralineata*–*C. lutheri* hybrids before hatching and (B) larvae immediately after hatching. All of the scale bars represent 0.5 mm. TT, pure *C. tetralineata*; LL, pure *C. lutheri*; and TL,*C. tetralineata*–*C. lutheri* hybrid.

To assess the allopatric of reproductive isolation, if any, between the two allopatric species and the reproductive ability of the individuals in the hybrid zone, mating tests were conducted via artificial fertilization with four experimental groups and two control groups. The development of eggs was recorded at several developmental stages in all of the experimental groups (Fig. 7). The time intervals between the selected developmental stages were similar among the experimental groups. Among the six characteristics examined, three characteristics of the estimated sizes of mature eggs, swelling eggs, and hatching larvae were significantly different among mating groups (*P *<* *0.001, Table 3). One common feature of the significantly different cases in Table S3 was that the differences occurred when the females of mating groups compared in pairwise were pure types of *C. lutheri* and *C. tetralineata*, respectively. For example, the mating group, TT ♀×LL ♂, showed significant differences with LL ♀×TT ♂ group but not with TT ♀×TT ♂ group. In contrast, there was no significant difference for the rest three characteristics of hatching, abnormality, and early survival rate (*P *>* *0.05, Table 3).

**Table 3:** Early life history characteristics of offspring from the mating experiments with six mating pairs.

	TT♀×LL♂	LL♀×TT♂	TL♀×TT♂	TL♀×LL♂	TT♀×TT♂	LL♀×LL♂	*P*
Replication	5	4	7	4	5	2	
Mature egg (mm)	1.11 ± 0.08	0.88 ± 0.09	1.01 ± 0.11	1.02 ± 0.22	1.04 ± 0.03	0.89 ± 0.03	<0.001*
Swelling egg (mm)	1.85 ± 0.14	1.62 ± 0.07	1.78 ± 0.08	1.77 ± 0.07	1.88 ± 0.04	1.65 ± 0.04	<0.001*
Hatching larva (mm)	4.74 ± 0.13	4.18 ± 0.10	4.21 ± 0.12	4.19 ± 0.17	4.55 ± 0.11	4.15 ± 0.22	<0.001*
Hatching rate (%)^1^	26.09	38.17	59.15	52.05	50.46	54.92	0.168
Abnormality rate (%)	2.88	12.75	12.48	4.22	4.96	0.51	0.406
Early survival rate (%)	97.08	86.76	87.17	95.18	94.87	99.25	0.384

The number of hatched individuals/the number of eggs.T, *Cobitis tetralineata* haploid; L, *C. lutheri* haploid; TL, *C. tetralineata*-*C. lutheri* hybrid.

*P*, probability of ANOVA among each group.

significant *P*-values (*P *<* *0.05) before Bonferroni correction.

## Discussion

### Allopatric speciation of two *Cobitis* species

The fishes belonging to the family Cobitidae are important in the freshwater ichthyofauna of the Korean peninsula, where 13 of 16 species from five genera have been reported as endemic species (Kim 2009). Our interested species, *Cobitis lutheri* and *C. tetralineata*, were known to be a sister group according to a gene tree based on Cyt *b* gene (Kim et al. 2002). Our calculation of the divergence time of them based on IMa2 analysis showed that they began to diverge approximately 3.3 MYA (95% HPD of 0.9–6.7) (Fig. 3B). In this analysis, the *C. lutheri* population of the Mangyeong River represented pure allopatric *C. lutheri* because morphologically and ecologically typical characteristics of this species were reported in that river (Kim and Lee 1988; Ko et al. 2009; Ko and Park 2011). Interestingly, the estimated divergence time of the two sister *Cobitis* species highly corresponded with the suspected disconnecting events of the Paleo-Yellow River in the northeast Asia in the late Pliocene (2.5–3.5 MYA) (see figure 46 in Nishimura 1974; Tada 1994). The vast areas of the East China Sea, the Yellow Sea between China and Korea, and near the sea of Kyushu of Japan locate on the shallow continental shelf of East Asia. Therefore, these shallow seas and their coastal lines should have been subject to wild fluctuations caused by both global and regional sea-level changes over geologic time. Tada (1994) suggested that the shallow southern Tsushima channel of the East Sea/Japan Sea began to reopen since the late Pliocene (2.5 MYA) and the Tsushima channel was closed for a very long time, about 2.5–10 MYA. This implies that the vast continental shelf of the northeast Asia might be exposed as land or slightly submerged by seawater before the late Pliocene. Other geologic records support the paleoceanographic conditions around that time period (Yi et al. 2003; Cukur et al. 2011). The wild changes of coastal lines were necessarily followed by connections and/or disconnections of the regional river systems. In this light of the link between sea-level changes and watercourses of rivers, the Korean river systems flowing westward and southward had been connected with the Paleo-Yellow River for a long time before the major paleoceanographic shift occurred around the late Pliocene (2.5–3.5 MYA) (see figure 46 in Nishimura 1974). We consider that the physical disconnection of the Korean river system by rising seawater might be associated with the allopatric splitting between *C. lutheri* and *C. tetralineata* around 3.3 MYA. The zero migration rates between them suggest that there was no postcontact between these *Cobitis* species to such an extent as to exchange gene pool since the disconnection of the paleo-Korean rivers, although the sea-level has been continuously oscillated by the cyclic glaciations since the Pleistocene. Another supporting evidence for the association between the evolution of *Cobitis* fish and the paleoceanographic changes in the late Pliocene comes from a divergence time estimation, 3.21 MYA, between two other sister species of Cobitidae, *Iksookimia hugowolfeldi* from the Youngsan River and *I. longicorpa* from the Seomjin River (Kim et al. 2013). We consider that the river disconnection events influenced by the sea-level changes around the late Pliocene could be served as a very important hypothetic framework for the evolutionary study of Korean freshwater fishes. Thus, a rigorous test for other taxonomic groups beyond Cobitidae is warranted.

### The genetic signature of the continuous introductions of non-native *C. tetralineata*

The closely related *Cobitis* species began to contact in the Dongjin River because of continuous and unidirectional introductions of the sister species *C. tetralineata* from the neighboring Seomjin River through two man-made canals. The haplotype diversity of the introduced *C. tetralineata* was relatively lower than that of native *C. lutheri* (Table 2). The star-like haplotype network of Ptr gene (Fig. 4) suggests a possibility of population expansion of the introduced population. As expected, the values of Tajima's *D* and Fu's *Fs* indicate a recent demographic expansion (*P *<* *0.005, Table S1). However, Cyt *b*, ENC1, and SH3PX3 did not show such significance (Table S1). Therefore, it appears that the significance of Ptr gene is caused by stochastic sampling variance of genetic locus or partly founder effect of the introduced individuals from a small source population. The gene pool of *C. tetralineata* in the Dongjin River has been maintained by its propagation in the non-native habitat and the constant supply of its original genomes from the Seomjin River as well. Genotypic assignments of the hybrid populations indicated that these introduced individuals originated from a single subpopulation of the Seomjin River, the Oknyeodong Stream (St. 5, Fig. 5b). The continuing gene flow from one source population must have contributed to the relatively less variable gene pool of the introduced *C. tetralineata* in the Dongjin River.

The presence of widespread hybridization between two closely related *Cobitis* species in the Dongjin River was initially indicated by morphological characteristics and allozyme markers (Kim and Yang 1993). Consistent with the previous results, our finding demonstrated an intensive genetic admixture between native *C. lutheri* and non-native *C. tetralineata* (Fig. 5). All the three groups within the hybrid zone showed very similar gradient patterns of admixture between two species (Fig. 5A), which reveals that hybridizations have been underway regardless of the microhabitats of the hybrid zone. The structure result (*K *=* *6) and pairwise *F*_ST_ between the nonhybridized allopatric populations of *C. lutheri* consistently showed apparent genetic differentiation among populations (Fig. 5B and Table S2). The high genetic differentiation reflects well the current physical isolation of the river systems flowing into the Yellow Sea. Nevertheless, a slight admixture between two distinct genetic group of *C. lutheri* was observed in the hybrid zone: one from the Youngsan River and the other from the Dongjin River. This result suggests that gene flow occurred between the two rivers in the recent past in contrast to the absence of exchange from the other northerly populations in the Geum and Mangyeong Rivers. As mentioned previously, sea-level changes had dramatically altered the coastlines of the Yellow Sea due to its shallowness (average water depth of 50 m) during the Pleistocene glacial periods (Ujiié and Ujiié 1999; Xiang et al. 2008). When the global sea-level reached approximately 120–130 m lower than the present level, the Yellow Sea completely disappeared. Thus, it is likely that the two southerly populations in the Youngsan and the Dongjin Rivers might be more closely connected than to the other northerly ones, when the sea-level went below the depth of the Yellow Sea during the glacial events.

The NewHybrids analysis allowed us to distinguish a range of different classes of hybrids, including F2 hybrids, backcrosses between parental individuals and F1 hybrids. As the result, six individuals in the hybrid zone were classified as pure *C. tetralineata* (P1) and others were identified as F2 hybrids (F2) or backcrossed individuals with *C. tetralineata* (B1), without F1 hybrids being observed (Fig. 6). To avoid confusion, it should be noted that the F2 hybrids do not literally mean the offspring of the crosses between F1 parents as usual as of the second generation of hybridization. On the contrary, the numerous F2 hybrids here correspond to the category of various post-F1 hybrids of successive hybridizations as such advanced hybrids beyond the second generation are highly likely assigned as F2 hybrids within the limit of two generation in the NewHybrids analysis (see figure 1 in Milne and Abbott 2008). Therefore, the F2 hybrids in Fig. 6 should be regarded as post-F1 hybrids because hybridizations have been occurring for 80 years in the Dongjin River.

The high proportion of post-F1 hybrids implies that the chance of mating with or between genetically pure parental individuals is low due to the accumulated increase of hybrid individuals descended from preceding hybrids, referred to as a hybrid swarm. This successful generation of a hybrid swarm appears to be the result of a lack of postzygotic barriers to reproduction between *C. lutheri* and *C. tetralineata*, despite the fact that this type of isolation is more commonly associated with allopatric species (Russell 2003). Our investigation of the postzygotic isolation between the two *Cobitis* species revealed that there is no discernable barrier. All of the experimental mating groups showed successful fertilization and development (Fig. 7), although three characteristics of the sizes of mature eggs, swelling eggs, and larvae were significantly different among the groups depending on the female species (Table 3). In addition, no significant difference in the other three viable rates after hatching was detected (Table 3). These results suggest that interbreeding between *C. lutheri* and *C. tetralineata* and backcrossing of their hybrids do not seem to lead to any effective disadvantages to their offspring. This interpretation is in accord with the explanation of hybrid swarm for the hybrid populations in the Dongjin River.

### Genomic replacement of native *C. lutheri* by introduced *C. tetralineata*

The present study indicates that the gene pool of native *C. lutheri* in the hybrid zone in Dongjin River has been profoundly changed by the creation of a hybrid swarm with its sister species *C. tetralineata*. Hereafter, we discuss the fate of this hybrid zone in both short- and long-term evolutionary aspects.

Hybrid populations have varying evolutionary consequences related to speciation and extinction, depending on the direction and intensity of natural selection (Harrison 1993; Huxel 1999; Barton 2001; Epifanio and Philipp 2001). In addition, hybrid swarms have been suggested to generate new genetic lineages or species, particularly when specific circumstances separating the hybrids from their parental species exist (Seehausen 2004; Mallet 2007; Abbott et al. 2013). For example, hybridization between *Cottus* fish species was found to have generated three distinct hybrid lineages through the formation of a hybrid swarm after secondary contact in the Rhine system, due to the presence of structured environments and high fecundity (Stemshorn et al. 2011). With respect to the selective direction of the *Cobitis* hybridization, neither genetic data nor mating experiments provide any information for it. Because the absence of evidence for any selective direction or trait does not necessarily prove the absence of it, we cannot rule out the possibility of such directional selection. Behavioral studies with mating experiments for multiple generations may be needed to detect it. As a more plausible explanation for the process and the fate of the hybrid zone, we focus on the effects of demographic processes and the environment of the Dongjin River.

Recently, a simulation study by Currat et al. (2008) gives insight into the mechanisms of introgression of genes between interbreeding species. They demonstrated that the direction and degree of introgression vary depending on two major factors, the strength of competition between two interacting species and demographic processes. Note that similar patterns of introgression could be yielded with or without invoking selection. The likelihood of having least introgression from local species to invasive species was consistently high when the population size of invasive species outnumbered that of local species regardless of the presence of competition or not (Currat et al. 2008). The situation of relatively high frequency of pure individuals of invasive species and backcrosses with it than those of the native species in the *Cobitis* hybrid zone seems to be analogous to the results of scenarios with larger population size of invasive species in Currat et al. (2008). We could not find any pure native *C. lutheri* and backcrossed hybrids with it (Fig. 6).

For two reasons, we think that the propagule pressure of non-native *C. tetralineata* may be larger than that of native *C. lutheri* in the hybrid zone in these days. Firstly, the introduction of *C. tetralineata* into the hybrid zone ever since 80 years ago still continues providing pure parental genomes from the Seomjin River to the hybrid zone. Secondly, the supply of pure native *C. lutheri* from other sources into the hybrid zone was effectively prevented by numerous low-head dams.

There are about 500 low-head dams distributed along the entire Dongjin River system for agricultural water catchment (Jang et al. 2010). These low-head dams act as barriers to free dispersals of fishes, particularly from the lower to upper parts of river tributaries, often leading to significant genetic differences in fish species between the lower and upper stretches of waterways (Ko et al. 2007, 2013). For this reason, it is strongly predicted that the native pure *C. lutheri* found in either the head stream beyond the exits of the water canals or other river tributaries diverging from the downstream of the main river (e.g., the Jeongeup Stream) are protected from the hybrid swarm by the low-head dams. If pure *C. lutheri* were to flow into the hybrid zone from the upper or lower region of the river, there would be continuous inputs of both pure *Cobitis* species into the hybrid zone of the river. This would, then, generate a more general hybrid zone structure that typically shows a cline of various traits across a geographically narrow zone between two parental species (e.g., Janko et al. 2005; Nolte et al. 2005; Macholán et al. 2007). However, *C. lutheri* was not observed in the upstream region of the Dongjin River (Shim and Kang 2009).

Thus, it appears that the original gene pool of the native *C. lutheri* has been replaced with that of non-native *C. tetralineata*, being influenced by the limited or absent inflow of genetically pure natives from the other tributaries. If we assume an absence of selective advantage to any of the *Cobitis* species, the rate of displacement through introgressions will be ultimately proportional to the amount of introduction of pure *C. tetralineata*. Genomic replacement of native species by introduced species has important implications for conservation in that such replacements could cause genomic extinction of pure natives, possibly within only a few generations (Wolf et al. 2001; Fitzpatrick et al. 2009, 2010; Ward et al. 2012). In fact, some instances of rapid replacement with invasive species through hybrid swarms have been reported. For example, the genetic composition of the native European honey bee (*Apis mellifera* L.) in the United States has been replaced to a considerable extent with that of introduced African honey bees (*A. mellifera scutellata*) through the creation of hybrid swarms in less than 50 years (Pinto et al. 2005; Kraus et al. 2007). The case of the native California tiger salamanders (*Ambystoma californiense*) also demonstrated such rapidity, as it took only approximately 20 generations for alleles from non-native tiger salamanders (*A. mavortium*) to become fixed in the genome of the pure population following the introduction of *A. mavortium* into the Salinas Valley in the United States (Fitzpatrick et al. 2009, 2010).

## Conclusion

The present study involving *Cobitis* hybrids between two sister species of native *C. lutheri* and introduced *C. tetralineata* in the Dongjin River, Korea, allowed us to understand the evolutionary consequences of the secondary contact brought about by the anthropogenic connection of waterways about 80 years ago. These two species were estimated to be split 3.3 million years ago (MYA) due to the disconnections of paleo-river systems driven by paleoceanographic changes in the northeast Asia in the late Pliocene. Population genetic analyses indicate that the native *C. lutheri* gene pool in the hybrid zone is likely being replaced by the introduced *C. tetralineata* gene pool via a hybrid swarm. The constant introductions of *C. tetralineata* from the neighboring Seomjin River through two water canals seem to greatly contribute to the rapid replacement of the original gene pool of *C. lutheri* in the hybrid zone. It is carefully anticipated that the genomes of native *C. lutheri* in the hybrid zone will ultimately go extinct unless the canals or low-head dams along the Dongjin River are eliminated. As the case of semi-isolated *C. lutheri*, secondary contacts and hybridizations between freshwater fish species that are abruptly brought about by man-made alterations will impact more greatly on small and isolated populations.

## Data Accessibility

All DNA sequences of four distinct genetic loci were deposited at Genbank popset (KF661552–KF661720, KF702400–703437), depending on loci.

## Supporting Information

Figure S1 Phylogenetic trees of haplotypes from the Dongjin River and nonhybridized allopatric populations of two *Cobitis* species of (A) Cytochrome *b*, (B) Ptr, (C) ENC1, and (D) SH3PX3 genes, based on unrooted neighbor- joining method. Dark and light gray shading rectangular boxes represent T-type and L-type group, respectively. Hybrid_Hap, Haplotypes from the Dongjin River; Lutheri_Hap, haplotypes from nonhybridized allopatric *C. lutheri* population; Tetralineata_hap, haplotypes from nonhybridized allopatric *C. tetralineata* population.

Figure S2 Histogram of the number of individuals sorted by the type of Cyt *b* gene and hybrid classes. F2, F2 hybrids; B1, backcrosses with pure *Cobitis tetralineata*; P1, pure *C. tetralineata*.

**Table S1.** Values of Tajima's *D* and Fu's *Fs*, and their *P*-values in four loci (Cyt *b*, ENC1, Ptr and SH3PX3 genes). **Table S2.** Pairwise *F*_ST_ values among the *Cobitis lutheri* and *C. tetralineata* populations from nonhybridized allopatric localities (locality 1, 2, and 3 for *C. lutheri*; locality 7 and 8 for *C. tetralineata*) using the multilocus genotypic data. All the *F*_ST_ values showed significant *P*-values (*P* < 0.001). **Table S3.***P*-values of Tukey's HSD in the post hoc analyses for the size of mature eggs, swelling eggs, and hatching larvae.
