# Supplementary material for: Genomic replacement of native Cobitis lutheri with introduced C. tetralineata through a hybrid swarm following the artificial connection of river systems
Source: Ecol Evol. 2014 Mar 24;4(8):1451–65. doi: 10.1002/ece3.1027 (PMC4020703; doi:10.1002/ece3.1027)
Supplement: Supplementary file 3 — Table S1. Values of Tajima's D and Fu's Fs, and their P-values in four loci (Cyt b, ENC1, Ptr and SH3PX3 genes). Table S2. Pairwise FST values among the Cobitis lutheri and C. tetralineata populations from nonhybridized allopatric localities (locality 1, 2, and 3 for C. lutheri; locality 7 and 8 for C. tetralineata) using the multilocus genotypic data. All the FST values showed significant P-values (P < 0.001). Table S3.P-values of Tukey's HSD in the post hoc analyses for the size of mature eggs, swelling eggs, and hatching larvae. [file ece30004-1451-sd3.docx]

Table S1. Values of Tajima’s *D* and Fu’s *Fs* and their *P*-values in four loci (Cyt *b*, ENC1, Ptr and SH3PX3 genes)

|  | Cyt *b* | | | ENC1 | | | Ptr | | | SH3PX3 | | |
| --- | --- | --- | --- | --- | --- | --- | --- | --- | --- | --- | --- | --- |
|  | L | T | T^D^ | L | T | T^D^ | L | T | T^D^ | L | T | T^D^ |
| Fu's *Fs* | -1.265 | - | -0.490 | 0.304 | 6.740 | 5.241 | -1.335 | -1.730 | -6.893 | -0.196 | -0.885 | -5.270 |
| *P* value | 0.344 | - | 0.384 | 0.599 | 0.976 | 0.950 | 0.244 | 0.035 | <0.001* | 0.499 | 0.179 | 0.031 |
| Tajima's *D* | -0.275 | 0.000 | -0.049 | 0.951 | 2.120 | 3.215 | -0.607 | -1.090 | -1.738 | -0.632 | -0.981 | 0.137 |
| *P* value | 0.451 | 1.000 | 0.521 | 0.839 | 0.978 | 0.998 | 0.330 | 0.118 | 0.005* | 0.315 | 0.076 | 0.594 |

L, *C. lutheri* in the Mangyeong River.

T, *C. tetralineata* in the Seomjin River

T^D^, T-type group of the Dongjin River.

* significant *P*-values (*P* < 0.02 for Fu’s *Fs*, *P* < 0.05 for Tajima’s *D*)

Table S2. Pairwise *F*_ST_ values among the *C. lutheri* and *C. tetralineata* populations from non-hybridized allopatric localities (locality 1, 2, and 3 for *C. lutheri*; locality 7 and 8 for *C. tetralineata* in figure 2) using the multi-locus genotypic data. All the *F*_ST_ values showed significant *P*-values (*P* < 0.001).

|  | locality 1 | locality 2 | locality 3 | locality 7 |
| --- | --- | --- | --- | --- |
| locality 2 | 0.191 |  |  |  |
| locality 3 | 0.183 | 0.128 |  |  |
| locality 7 | 0.305 | 0.168 | 0.239 |  |
| locality 8 | 0.364 | 0.236 | 0.282 | 0.129 |

Table S3. *P*-values of Tukey’s HSD in the post hoc analyses for the size of mature eggs, swelling eggs, and hatching larvae.

| Mature  eggs (mm) | TT♀×LL♂ | LL♀×TT♂ | TL♀×TT♂ | TL♀×LL♂ | TT♀×TT♂ | LL♀×LL♂ |
| --- | --- | --- | --- | --- | --- | --- |
| TT♀×LL♂ |  |  |  |  |  |  |
| LL♀×TT♂ | <0.001* |  |  |  |  |  |
| TL♀×TT♂ | 0.219 | 0.031* |  |  |  |  |
| TL♀×LL♂ | 0.200 | 0.008* | 1.000 |  |  |  |
| TT♀×TT♂ | 0.602 | <0.001* | 0.961 | 0.973 |  |  |
| LL♀×LL♂ | <0.001* | 0.998 | 0.071 | 0.022* | 0.001* |  |
| Swelling  eggs (mm) | TT♀×LL♂ | LL♀×TT♂ | TL♀×TT♂ | TL♀×LL♂ | TT♀×TT♂ | LL♀×LL♂ |
| TT♀×LL♂ |  |  |  |  |  |  |
| LL♀×TT♂ | <0.001* |  |  |  |  |  |
| TL♀×TT♂ | 0.318 | <0.001* |  |  |  |  |
| TL♀×LL♂ | 0.097 | <0.001* | 0.999 |  |  |  |
| TT♀×TT♂ | 0.892 | <0.001* | 0.032* | 0.004* |  |  |
| LL♀×LL♂ | <0.001* | 0.582 | 0.001* | 0.001* | <0.001* |  |
| Hatching  larvae (mm) | TT♀×LL♂ | LL♀×TT♂ | TL♀×TT♂ | TL♀×LL♂ | TT♀×TT♂ | LL♀×LL♂ |
| TT♀×LL♂ |  |  |  |  |  |  |
| LL♀×TT♂ | <0.001* |  |  |  |  |  |
| TL♀×TT♂ | <0.001* | 0.998 |  |  |  |  |
| TL♀×LL♂ | <0.001* | 1.000 | 1.000 |  |  |  |
| TT♀×TT♂ | 0.074 | <0.001* | <0.001* | <0.001* |  |  |
| LL♀×LL♂ | <0.001* | 0.997 | 0.952 | 0.988 | <0.001* |  |

* significant *P*-values (P < 0.05) before Bonferroni correction.
